# Interactive live-stream surgery contributes to surgical education in the context of contact restrictions

**DOI:** 10.1007/s00405-021-06994-0

**Published:** 2021-08-23

**Authors:** Sara M. van Bonn, Jan S. Grajek, Armin Schneider, Tobias Oberhoffner, Robert Mlynski, Nora M. Weiss

**Affiliations:** 1 Department of Otorhinolaryngology, Head and Neck Surgery, Otto Körner”, Rostock University Medical Center, Doberaner Strasse 137-139, D-18057 Rostock, Germany; 2 Munich Surgical Imaging GmbH, Türkenstraße 89, 80799 Munich, Germany; 3 Research Group Minimally Invasive Interdisciplinary Therapeutical Intervention (MITI), KlinikumRechts Der Isar”, Technical University Munich (TUM), Munich, Germany

**Keywords:** E-Learning, Medical education, Live surgery, Otorhinolaryngology, Communication, COVID-19

## Abstract

**Background:**

Attendance teaching is the predominant teaching method at universities but needs to be questioned in the context of digital transformation. This study establishes and evaluates a method to accomplish electronic learning to supplement traditional attendance courses.

**Materials and methods:**

Surgery was transmitted in real-time conditions via an online live stream from the surgical theater. Visualization was transferred from a fully digital surgical microscope, an endoscope or an environmental camera in high definition quality. Students were able to participate at home from their personal computer. After following the surgery, they participated in an online-evaluation.

**Results:**

A total of 65 students participated in the live stream. The majority of students (61.54%) indicated a significant subjective increase in knowledge after participation. The majority of students (53.85%) indicated that live surgeries should be offered as a permanent component in addition to classroom teaching. Likewise, a broader offer was desired by many students (63.08%).

**Conclusions:**

Live streaming of surgery is a promising approach as an alternative or supplement to traditional attendance teaching. An expansion of digital teaching can be explicitly supported on the basis of this study.

## Introduction

Electronic learning (e-learning) is a comprehensive concept for self-determined and computer-supported teaching and learning processes [1]. Teaching materials that are available electronically have become increasingly important in recent years [2, 3]. E-learning offers the possibility to use different terms of visualization. Another advantage is that learning may be individualized to personal needs and learning requirements and also offers a large flexibility since it is not locally bound [4, 5].

In the context of the current Covid-19 pandemic, e-learning has gained additional importance due to the limitations of attendance teaching. The evolution from analog to digital teaching has accelerated [6, 7]. Universities have been confronted with significant challenges to provide regular teaching online in a very short transition period. In the graduate medical education environment, this circumstance was further complicated by the requirement to provide practical and theoretical instructions at the patient's bedside [8]. When testing new innovative teaching materials and methods the evaluation of subjective benefits in addition to objective procedures, such as exams, is also important  [9, 10].

In surgical subjects, anatomical knowledge and anatomical training is provided by lectures using various forms of presentation (e.g. videos, images) but still, attendance courses in the surgical theater with an interaction between students and surgeon play a major role. Consequently, these skills and methods are particularly neglected in distance learning.

Recent reviews show that digital teaching formats have great potential for medical education and are not inferior to traditional lectures [9, 11]. Interactive computer-based methods combined with hands-on teaching have shown great potential in general anatomy education [7, 12]. Furthermore, it has been described that medical students using electronic learning programs are subjectively more satisfied with their learning experience than students using traditional learning methods [4, 13]. However, the advantages of attendance teaching may not be waived in medical education since they offer the opportunity to communicate on the current content of the lesson, e.g. the surgical steps and specialties. For this reason, transforming the advantage of practical surgical courses into a digital context is challenging but promising for future educational concepts.

For this reason, it was the aim of this study to establish and to evaluate interactive live surgery as a new method for anatomical and surgical teaching in the context of contact restrictions that may be included into e-learning programs.

## Methods

Prior surgery, patients gave written informed consent for the anonymized transmission of surgical imaging for educational purposes.

**Fig. 1:**
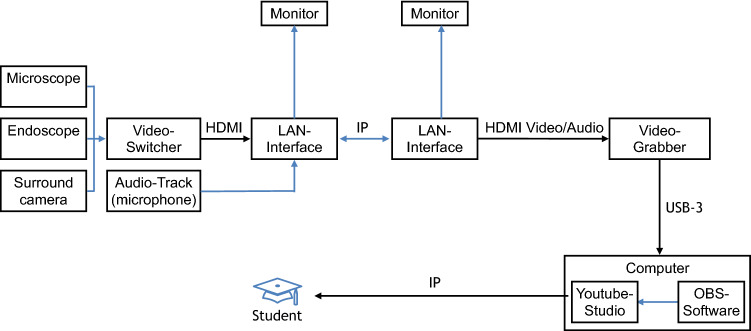
Scheme of the transmission setup

A live stream in high definition (HD) quality from the surgical theater was offered to students from 7th and 8th year on several days by prior announcement. Within the surgical theater, surgery and surroundings were recorded by a fully digital surgical microscope (Arriscope Evo2 ENT, MSI, Munich, Germany), standard functional endoscopic sinus surgery endoscopes with HD camera (Aida, Storz, Tuttlingen, Germany), and an environmental camera (EVI-HD7V, Sony, Tokyo, Japan), respectively. The surgeon was equipped with a digital wireless bodypack sound transmitter (Shure GLXD1, Niles, USA) that was connected to a rack mount receiver (Shure GLXD4R, Niles, USA). The microphone was sensitive enough to transmit the intraoperative discussions and decision making of the entire team to the students. Audio track from the rack mount receiver as well as the video track from the surgical microscope and the endoscope were connected with a broadcast switcher (ATEM Mini, Blackmagic Design Pty Ltd, Victoria, Australia) that was linked to a conferencing room system (Avaya Scopia XT7100, Avaya, Santa Clara, CA, USA). Audio track und video footage were transferred to a media service provider (Pripares, Munich, Germany) via Local area network (LAN) interface. The data was transformed by the service provider and then transferred online in 4 K-high-definition quality with a chat function via a dedicated channel on YouTube (Google LLC, Mountain View, USA) alternatively on Vimeo (Vimeo LLC, NY, USA). Figure 1 shows the transmission setup. Students were able to access the stream from any location in Germany as well as worldwide via common terminals after receiving an individual and daily changing password. The students were able to ask questions concerning the surgical procedures via a chat function. The questions were passed by one of the staff members to the surgeon and answers were given by the surgeon so that they could be heard and discussed by the complete audience. However, since the students were muted and questions were only asked via the chat function, a disruption of the surgeon and the surgical team was avoided and the discussion could be terminated to less critical parts of the surgery. In this pilot-study various ear, nose and throat (ENT) surgeries were transmitted to the students.

At the end of the live surgery, students were asked to complete an evaluation questionnaire provided on the online platform ILIAS (Table 1). The open-source product ILIAS is a free hypertext transfer protocol that is used by many universities (ILIAS open-source e-Learning e.V., Cologne, Germany) for the internet-based provision of teaching and learning materials. The first eight questions had to be answered on a 5-point Likert scale (full agreement = 1 points; agreement = 2 points; indecisive = 3 points; rather no agreement = 4 points; no agreement = 5 point, no response possible = 6 points), six statements  had to be evaluated by a rating scale with gradings from very good to deficient and two questions were open-ended for response.

**Table 1:** Evaluation questionnaire after participation in a live surgery

Evaluation questionnaire (Response options: “full agreement”, “agreement”, “indecisive”, “rather no agreement”, “no agreement” and the option “no response possible”)
1 The live streaming surgeries gave me a great increase in knowledge
2 Live surgeries should be an integral part of teaching
3 The live video format offers a good alternative to traditional surgical teaching
4 The offer of live surgeries at the University Medical Center Rostock should be increased
5 My hardware (tablet, PC, cell phone, etc.) was able to replay the live video without any problems
6 The live-chat module was a useful way to interact with the surgeon
7 The obligation to have a Google account to use the chat function was annoying
8 I would agree to pay money to follow the live surgeries
Rating scale (Response options “very good”, “good”, “satisfactory”, “sufficient”, “non-sufficient”, “deficient” and the option “no response possible”)
9 Video transmission quality
10 Audio transmission quality
11 Stability of the connection to the channel
12 Comprehensibility of intraoperative processes
13 Possibility of interaction with the surgeon
Open questions
14 I used the following hardware during the live stream (e.g. cell phone, Tablet, Computer, Laptop)
15 I used the following browser for streaming (e.g. Firefox, Opera, MS-Explorer, Safari, Google Chrome)

Statistical analyses were performed using Prism (version 8, GraphPad Software, La Jolla, CA, USA). If not otherwise specified, data are presented as median with 95% confidence interval (95%CI) or as absolute numbers with percentages

## Results

**Fig. 2:**
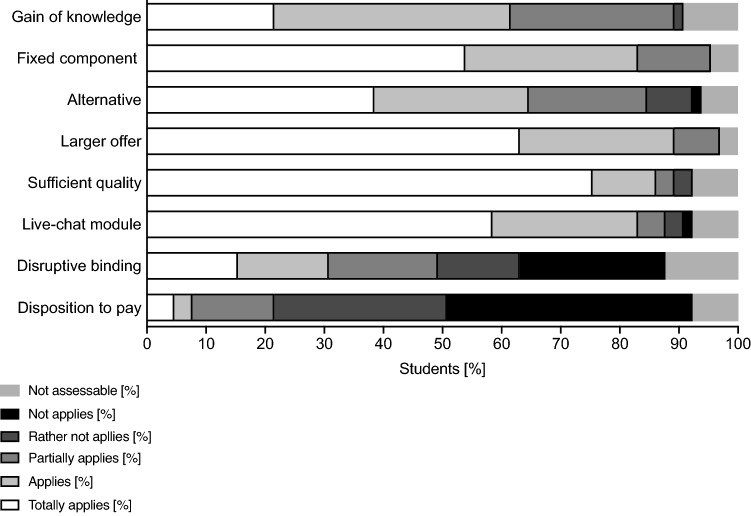
Answers to the evaluation questions of statement 1 to 8

65 students participated in the live surgeries and completed the evaluation. 64 students completed the rating scale. The evaluation questionnaires are shown in Table 1. The descriptive statistic responses to the questionnaires are shown in Table 2 and Table 3. A total of 61.5% of the students reported a subjective gain of knowledge after watching the live-stream (“totally applies”, “applies”), 83% stated, that live-surgery should be an integral part of teaching and 64.6% stated, that the live-stream offers an alternative to traditional teaching methods. Only 3% reported a rather insufficient quality and 89.2% asked for an increased offer of live-stream surgery. None of the students rated either the quality of audio, video, the stability of the connection or the comprehensibility of the intraoperative processes as insufficient. Only 3% considered the interactivity inadequate. The answers from the students to the evaluation questions are shown in Fig. 2, 3. To participate in the live surgery, 46 students (71.88%) used a laptop, 9 students (14.06%) a tablet, 7 students (10.94%) a computer and 2 students (3.13%) a mobile phone. As internet browser, 24 students (37.5%) used Google Chrome, 23 students (35.94%) used Safari, 12 students (18.75%) used Firefox, 4 students (6.25%) used Microsoft Edge and one student (1.56%) used Ecosia.

**Table 2:** Descriptive statistics of statement 1 to 8

Statement	1	2	3	4	5	6	7	8
Number of values	65	65	65	65	65	65	65	65
Minimum	1	1	1	1	1	1	1	1
25% percentile	2	1	1	1	1	1	2	4
Median	2	1	2	1	1	1	4	4
75% percentile	3	2	3	2	1.5	2	5	5
Maximum	6	6	6	6	6	6	6	6
Range	5	5	5	5	5	5	5	5

**Table 3:** Descriptive statistics of statement 9 to 13

Statement	9	10	11	12	13
Number of values	64	64	64	64	64
Minimum	1	1	1	1	1
25% percentile	1	1.25	2	2	1
Median	2	2	2	2	2
75% percentile	2	2	3	3	3
Maximum	4	4	4	4	5
Range	3	3	3	3	4

**Fig. 3:**
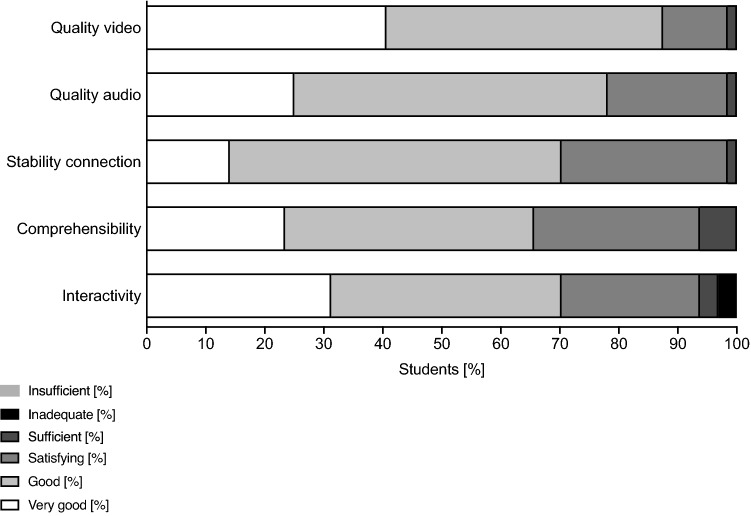
Answers to the evaluation questions of statement 9 to 13

## Discussion

Digital transformation of teaching has gained importance within the last years. Digital learning formats have even been politically promoted by the Federal Ministry of Education and Research in Germany in recent years. An improved digital infrastructure enabling a better knowledge exchange is desirable [14]. However, according to an analysis conducted by Saß et al., in which 18 departments and 29 teaching staff members from German university hospitals were surveyed regarding the use of e-learning in the curriculum of otolaryngology, a low level of implementation was found regardless of the location [15].

To ensure student training even under contact restrictions due to the ongoing Covid-19 pandemic, a digital teaching offer had to be created within the last year [4, 16]. E-learning-based teaching approaches currently show a growing popularity and are increasingly focused in university medical education [15]. This study attempted to present a method of broad and comprehensive assessment of surgical contents by the creation and transmission of high-definition video material with the opportunity of interaction between students and surgeon.

In a recent study, a high demand for additional instruction and teaching content especially for audio-material and videos is reported [17]. With the opportunity to ask questions and receive immediate response, in this study, the participation was even more interactive compared to passive viewing of recorded videos only. Thus, the setup may improve surgical education and explanation of special procedures. Since the majority of participants reported an increase in knowledge, an integration of the presented method into digital learning may be recommended.

The use of digital elements in medical education not only supports traditional teaching, but also offers a variety of possibilities for medical didactics. Innovative teaching components such as live-surgery contribute to flexible and user-orientated learning strategies. The superiority of electronic learning programs over traditional teaching methods has been discussed, especially with regard to internationally uniform standards [18]. An equivalent effectiveness and efficiency of traditional teaching and modern digital formats is assumed [19–21].

The visualization and transmission of surgical content may also be applied in other surgical specialties and is an interesting supplement for surgical education that even enables a temporary substitution of presence. The opportunity to reach many students without disruptions in the surgical theatre is considered an advantageous innovation in the education of medical students. The established setup may also be applied to demonstrate preparation procedures from anatomical courses, the demonstration of radiologic imaging and may provide additional explanation from nursing or physiotherapy. Furthermore, a larger number of students can be reached compared to clinical courses with presence in clinics, surgical theaters or classrooms. The students show a high level of interest in modern concepts for the implementation and further development of digitalization in teaching and positive acceptance of the topic [19]. Consequently, a good motivation with regular use of the format may be expected.

Future learning concepts should be able to offer the opportunity to be used digital, mobile and accessible at any time. Although classic learning methods such as lectures and practical courses cannot entirely be replaced by electronic media, they are increasingly used in combination with each other [17, 22]. The presented setup has great potential, for future application even after the Covid-19 pandemic. Direct feedback and mentoring are possible despite contact restrictions. These innovations may also be used to expand interdisciplinary or international co-education and collaboration, especially in terms of sustainability, resource conservation or even development aid. The international exchange of expertise, especially in remote areas with limited resources is an important basis for scientific collaborations and the transfer of knowledge. In this case, live streaming of surgeries is considered as a complementary teaching tool. Especially the combined use of surgical microscopes, endoscopes, as well as surrounding cameras provide promising opportunities to enrich surgical training. Developments in the field of electronic learning may contribute to a revolution in the education system in the future [11, 15, 23].

The method is limited by the requirement of stable internet connection. However, the quality of the video and audio transmission as well as the stability of the connection was consistently rated as good by the students. Furthermore, the study is limited by the fact that it is not possible to draw conclusions about the additional learning success by the use of live-stream surgery. Recording learning success depends, among other things, on the individual knowledge of the respective students. However, the presented study was rather designed in the sense of a methodological approach. Future studies should be dedicated to the individual gain of knowledge using digital teaching methods and live surgery.

## Conclusions

The results of this study show that live streaming of surgical procedures is a promising approach as supplement to traditional learning. It is a valuable alternative approach for surgical education, especially for the application of already acquired anatomical knowledge. It enables an approximation to attendance courses despite contact restrictions. An implementation of this method into digital teaching is recommended.

## References

[1] Clark RC, Mayer RE (2016). E-learning and the science of instruction: proven guidelines for consumers and designers of multimedia learning.

[2] Offergeld C, Neudert M, Emerich M, Schmidt T, Kuhn S, Giesler M (2020). Mediation of data literacy in curricular education in otorhinolaryngology: watch and wait or anticipatory obedience?. HNO.

[3] Gaupp R, Fabry G, Körner M (2018). Self-regulated learning and critical reflection in an e-learning on patient safety for third-year medical students. Int J Med Educ.

[4] Tarpada SP, Hsueh WD, Gibber MJ (2017). Resident and student education in otolaryngology: a 10-year update on e-learning. Laryngoscope.

[5] Vaona A, Banzi R, Kwag KH, Rigon G, Cereda D, Pecoraro V (2018). E-learning for health professionals. Cochrane Database Syst Rev.

[6] Stöver T, Dazert S, Hoffmann TK, Plontke SK, Zenk J (2020). Effects of the SARS-CoV-2 pandemic on the otorhinolaryngology university hospitals in the field of medical care. Laryngorhinootologie.

[7] Weiss NM, Schneider A, Hempel JM, Uecker FC, van Bonn SM, Schraven SP (2020). Evaluating the didactic value of 3D visualization in otosurgery. Eur Arch Oto-Rhino-Laryngol.

[8] Offergeld C, Ketterer M, Neudert M, Hassepaß F, Weerda N, Richter B (2020). “Online from tomorrow on please”: comparison of digital framework conditions of curricular teaching at national university ENT clinics in times of COVID-19: digital teaching at national university ENT clinics. HNO.

[9] Faiz T, Marar O, Kamel MK, Vance S (2020). Teaching operative surgery to medical students using live streaming during COVID-19 pandemic. Surg Innov.

[10] Bechstein M, Elsheikh S, Wodarg F, Taschner CA, Hanning U, Buhk J-H (2020). Interhospital teleproctoring of endovascular intracranial aneurysm treatment using a dedicated live-streaming technology: first experiences during the COVID-19 pandemic. BMJ Case Rep.

[11] Jack MM, Gattozzi DA, Camarata PJ, Shah KJ (2021). Live-streaming surgery for medical student education—educational solutions in neurosurgery during the COVID-19 pandemic. J Surg Educ.

[12] Stanford W, Erkonen WE, Cassell MD, Moran BD, Easley G, Carris RL, Albanese MA (1994). Evaluation of a computer-based program for teaching cardiac anatomy. Invest Radiol.

[13] Funke K, Bonrath E, Mardin A, Becker C, Haier J, Senninger N (2013). Blended learning in surgery using the inmedea simulator. Langenbeck Arch Surg.

[14] BMBF-Internetredaktion. Digital media in vocational training - BMBF. 2019. https://www.bmbf.de/de/digitale-medien-in-der-bildung-1380.html. Accessed 21 Mar 2021.697Z.

[15] von Saß PF, Klenzner T, Scheckenbach K, Chaker A (2017). E-learning in ENT: usage in university medical centers in Germany. Laryngorhinootologie.

[16] Güzer B, Caner H (2014). The past, present and future of blended learning: an in depth analysis of literature. Procedia Soc Behav Sci.

[17] Shabli S, Heuermann K, Leffers D, Kriesche F, Abrams N, Yilmaz M (2019). Survey on the need for an e-learning-platform for ENT residents. Laryngorhinootologie.

[18] Lau F, Bates J (2004). A review of e-learning practices for undergraduate medical education. J Med Syst.

[19] Wormald BW, Schoeman S, Somasunderam A, Penn M (2009). Assessment drives learning: an unavoidable truth?. Anat Sci Educ.

[20] Ikonne U, Campbell AM, Whelihan KE, Bay RC, Lewis JH (2018). Exodus from the classroom: student perceptions, lecture capture technology, and the inception of on-demand preclinical medical education. J Am Osteopath Assoc.

[21] Stanford W, Erkonen WE, Cassell MD, Moran BD, Easley G, Carris RL, Albanese MA (1994). Evaluation of a computer-based program for teaching cardiac anatomy. Invest Radiol.

[22] Koch GK, Sethi RKV, Kozin ED, Bergmark RW, Gray ST, Metson R (2017). Online teaching tool for sinus surgery: trends toward mobile and global education. OTO Open.

[23] von Sass PF, Scheckenbach K, Wagenmann M, Klenzner T, Schipper J, Chaker A (2015). Taking a fresh look at the skull base in otorhinolaryngology with web-based simulation: student’s interactive skull-base trainer (SISTer). JAMA Otolaryngol Head Neck Surg.

